# Supplementary figures and images for: Fine Tuning of Calcium Constitutive Entry by Optogenetically-Controlled Membrane Polarization: Impact on Cell Migration
Source: Cells. 2020 Jul 13;9(7):1684. doi: 10.3390/cells9071684 (PMC7408270; doi:10.3390/cells9071684)

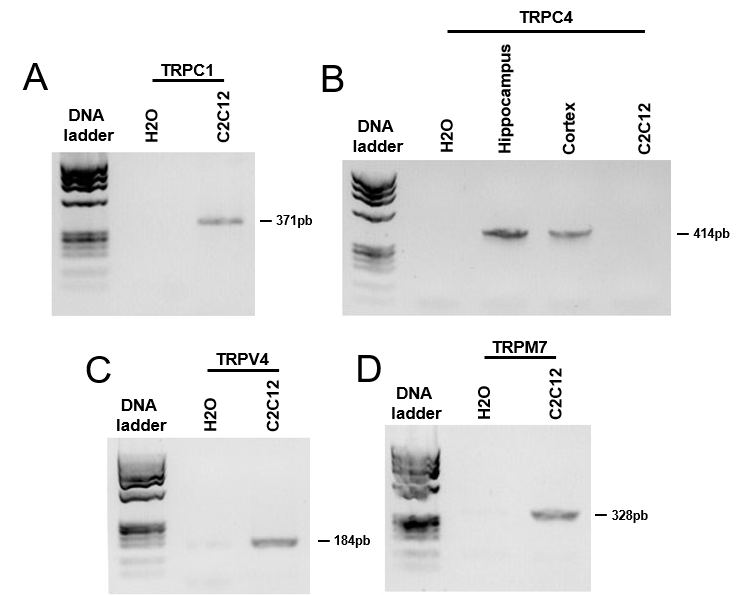

Supplement: Supplementary file 1 [file cells-09-01684-s001.zip › Figure S1.tif]
